# Closed-loop brain-computer interfaces for post-stroke sensorimotor loop restoration

**DOI:** 10.3389/fnins.2026.1882653

**Published:** 2026-08-20

**Authors:** Ran Tao, Can Duan, Yang-Pu Zhang

**Affiliations:** 1 School of Acupuncture-Moxibustion and Orthopedics, Hubei University of Chinese Medicine, Wuhan, China; 2 Hubei Provincial Hospital of Integrated Traditional Chinese and Western Medicine, Wuhan, China; 3 Hubei Provincial Clinical Research Center for Stroke Rehabilitation of Integrated Traditional Chinese and Western Medicine, Wuhan, China

**Keywords:** closed-loop brain–computer interface, neuroplasticity, precision neurorehabilitation, sensorimotor loop, stroke rehabilitation

## Abstract

Stroke recovery is increasingly understood as a process shaped by disrupted interactions among motor intention, descending motor output, peripheral movement, and sensory feedback, rather than by motor weakness alone. After stroke, residual motor intention may not be effectively translated into spinal motor output, peripheral movement may be too limited to provide sufficient sensory feedback, and compensatory network recruitment may not always support efficient motor control. Closed-loop brain-computer interfaces offer a mechanistically motivated approach to this problem by detecting motor imagery, motor attempt, or sensorimotor rhythms in real time and translating these signals into contingent functional electrical stimulation, robotic assistance, virtual reality, multisensory feedback, or neuromodulation. By partially restoring the temporal coupling between detected motor intention, assisted or stimulated movement, and sensory feedback, these systems are hypothesized to promote activity-dependent plasticity, reinforce sensorimotor circuits, and modulate distributed motor, sensory, attentional, and interhemispheric networks. In this review, we synthesize the neurobiological basis, recovery mechanisms, implementation strategies, and emerging predictive biomarkers of closed-loop brain-computer interfaces for post-stroke sensorimotor rehabilitation. Although closed-loop brain-computer interfaces show promise for selected patients, treatment effects remain heterogeneous and depend on patient characteristics, decodability of motor-related brain signals, intervention dose, feedback modality, and trial design. Future progress will require mechanism-informed patient stratification, adaptive decoding, individualized feedback, standardized outcomes, and real-world validation.

## 1 Introduction

Stroke remains a major cause of death and long-term disability worldwide, and many survivors experience persistent motor, sensory, cognitive, and participation-related impairments (Feigin et al., 2021, 2025; Martin et al., 2025). Upper-limb sensorimotor dysfunction is especially important because it limits grasping, manipulation, self-care, and social participation, thereby affecting independence and rehabilitation needs (O'Flaherty et al., 2024). Although current rehabilitation guidelines emphasize early, intensive, task-specific, and repetitive practice, upper-limb recovery remains highly variable, and many patients do not regain sufficient function after the subacute phase (Kolmos et al., 2015). Therefore, rehabilitation strategies should not only increase practice intensity but also help couple residual motor intention with movement-related sensory feedback.

Post-stroke motor impairment is not simply a consequence of muscle weakness; rather, it reflects the combined effects of corticospinal output disruption, impaired sensory feedback, higher-order sensorimotor integration deficits, and interhemispheric imbalance (Campos et al., 2023; Hoh and Semrau, 2025; Sahrizan et al., 2025; Chen et al., 2023). Effective movement depends on corticospinal integrity, sensory input, motor-network connectivity, and adaptive compensatory reorganization. Conventional therapy largely relies on visible movement and therapist-guided practice. However, in patients with severe paresis, sensory loss, or insufficient voluntary output, motor intention, muscle activation, and sensory feedback are often temporally and quantitatively decoupled, limiting activity-dependent plasticity. Reconstructing the sensorimotor loop represents a critical entry point for understanding recovery mechanisms and developing neuromodulatory rehabilitation strategies.

Brain-computer interfaces (BCIs) decode motor imagery, motor attempt, or sensorimotor rhythms and transform these neural signals into functional electrical stimulation (FES), robotic assistance, virtual reality (VR) feedback, non-invasive brain stimulation (NIBS), or other external outputs, thereby enabling a shift from passive training to brain-state-driven rehabilitation (Mansour et al., 2022). Here, closed-loop refers to systems in which therapeutic output is adjusted according to the patient's real-time neural state, commonly recorded by electroencephalography (EEG), rather than fixed timing or preset programs. In this review, NIBS includes transcranial direct current stimulation (tDCS) and repetitive transcranial magnetic stimulation (rTMS), and the primary motor cortex is abbreviated as M1. Adaptive BCI refers to systems that update decoding or feedback parameters according to neural or behavioral changes. Recent randomized controlled trials and meta-analyses suggest that BCI-based interventions, particularly when combined with FES or embodied feedback, may improve upper-limb motor function, cortical activation, and neuroplasticity-related outcomes (Wang et al., 2024; Brunner et al., 2024; Ma et al., 2024; Ren et al., 2024; Kim et al., 2025). Therefore, closed-loop BCI is reviewed here as a mechanistic intervention rather than only as an assistive technology.

A key limitation is that clinical efficacy evidence remains insufficiently integrated with mechanistic findings, and unified frameworks for patient stratification, feedback optimization, and home-based deployment are still lacking (Jin et al., 2024; Liu J. et al., 2025; Chen and Yun, 2026; Guo et al., 2026). This review summarizes the neurobiological basis of post-stroke sensorimotor loop disruption, the mechanisms and implementation modes of closed-loop BCI, and emerging biomarkers and translational strategies for efficacy prediction and patient stratification.

This review was designed as a narrative translational synthesis. PubMed, Web of Science, Scopus, and Google Scholar were searched for English-language studies published up to May 2026 on closed-loop BCI, stroke rehabilitation, sensorimotor recovery, neuroplasticity, implementation modes, biomarkers, and home-based rehabilitation. The search was conducted iteratively and refined according to emerging mechanistic, clinical, and translational themes. Reference lists of key reviews and eligible studies were also screened to identify additional relevant publications, and duplicate records were removed before study assessment. Study relevance and inclusion were evaluated according to the predefined scope of the review and confirmed through discussion among the authors. Randomized controlled trials, systematic reviews, meta-analyses, and human experimental studies were prioritized for clinical efficacy, while technical, feasibility, and mechanistic studies were included when relevant to biological rationale, patient selection, device implementation, or translational challenges. The review was restricted to non-invasive, rehabilitation-oriented closed-loop BCI approaches, which currently form the main clinical evidence base in post-stroke rehabilitation. Invasive BCI systems were outside the scope of this review, which represents a limitation given their different signal quality, risk profile, ethical requirements, and translational pathway.

## 2 Neurobiological basis of post-stroke sensorimotor loop disruption

**Figure 1:**
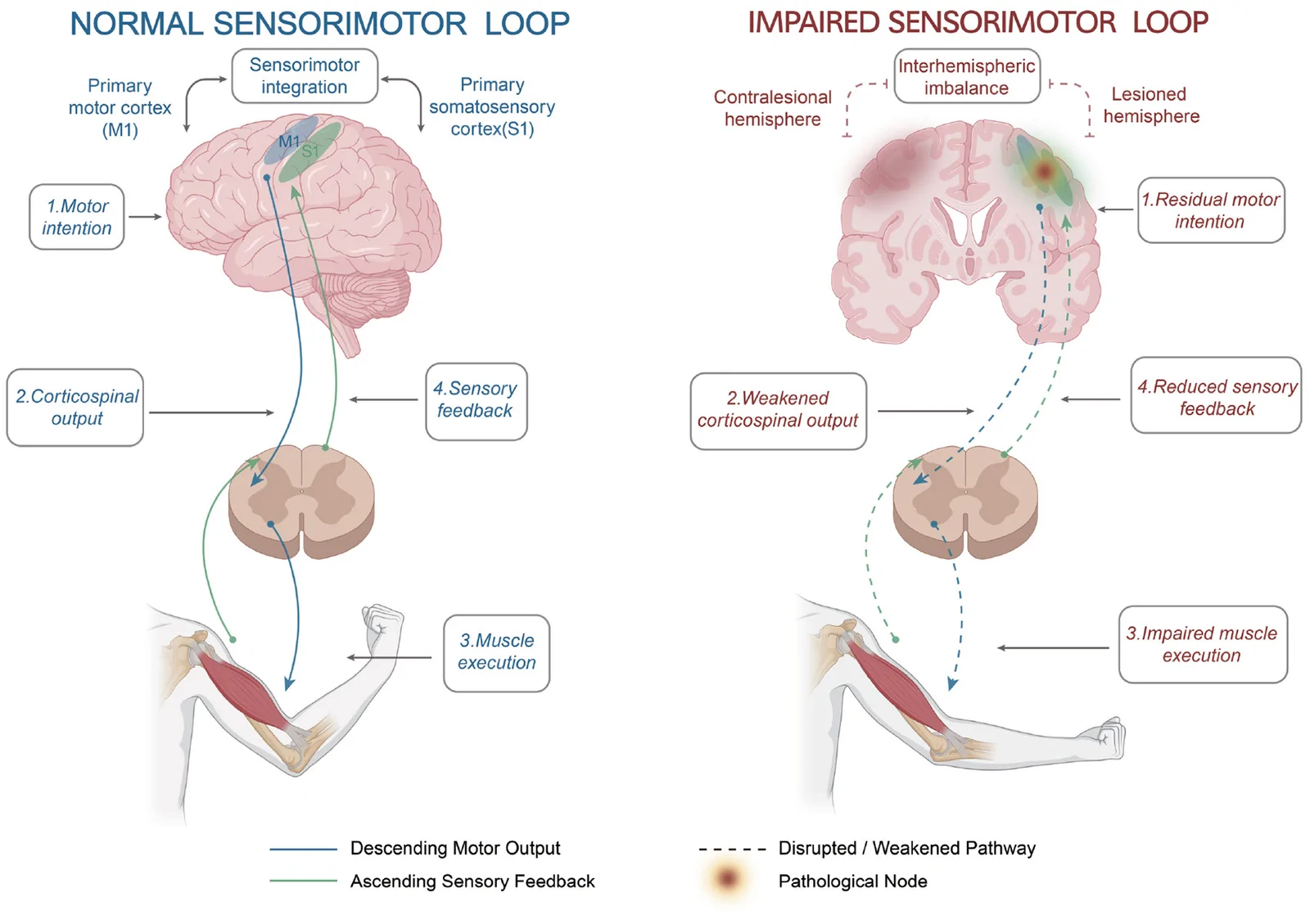
Neurobiological framework of post-stroke sensorimotor loop disruption. The left panel shows sensorimotor integration between the primary motor cortex and primary somatosensory cortex in a lateral brain view. The right panel shows the lesioned and contralesional hemispheres in a coronal view, highlighting interhemispheric imbalance after stroke. The primary motor and somatosensory cortices related to the affected limb are shown within the same lesioned hemisphere. Stroke-related injury disrupts motor intention, corticospinal output, peripheral execution, sensory feedback, and higher-order sensorimotor integration, contributing to impaired voluntary movement and heterogeneous recovery. This framework integrates current evidence on post-stroke sensorimotor disruption, although the causal relationships among its components remain to be fully established.

Before discussing each pathological component, this section summarizes the neurobiological framework of post-stroke sensorimotor loop disruption. As shown in Figure 1, stroke-related injury disrupts the transformation from motor intention to descending output, peripheral execution, sensory feedback, and higher-order sensorimotor integration. Corticospinal damage, sensory feedback loss, and interhemispheric reorganization jointly impair the closed sensorimotor loop supporting voluntary movement and recovery. Although this framework focuses on cortical, corticospinal, sensory, and interhemispheric mechanisms, recent evidence also supports the contribution of subcortical and cerebellar circuits to post-stroke motor recovery.

### 2.1 Impaired corticospinal output and disturbed execution of motor intention

The corticospinal tract is the main descending pathway linking motor cortical activity to spinal motor output, and its integrity is essential for translating motor intention into selective muscle recruitment and fine motor control. Neuroimaging studies show that corticospinal white-matter integrity is closely related to chronic upper-limb function, although a single corticospinal measure cannot fully explain functional heterogeneity in severely impaired patients (Domin et al., 2023; Hayward et al., 2022). This suggests that residual descending pathways, interhemispheric connectivity, and whole-brain structural reserve may also influence recovery. Pathway-specific studies further indicate that corticospinal damage is closely associated with impaired distal control and motor skill acquisition, whereas alternative descending systems cannot fully restore the precise cortical output needed for dexterous movement (Paul et al., 2023a).

Corticospinal deficits are also task-specific and network-dependent. Diffusion imaging studies suggest that M1-derived corticospinal fibers mainly support strength and simple joint control, whereas fibers from premotor and supplementary motor areas contribute more to complex motor control, inter-joint coordination, and visuomotor integration (Paul et al., 2024). Connectome-based lesion analyses show that different motor tasks are associated with distinct disconnection patterns, indicating that global upper-limb scales may obscure task-specific anatomical differences (Esser et al., 2025). Pre-existing white-matter hyperintensities may further reduce structural reserve and influence motor outcomes after corticospinal injury (Ferris et al., 2024). Neurophysiologically, TMS-derived motor evoked potentials provide useful information on corticospinal transmission, but single-muscle resting responses cannot fully represent multi-segmental upper-limb control; task-state, multi-muscle, and proximal-distal assessments may therefore be needed (Kumar et al., 2024).

### 2.2 Loss of sensory feedback and impaired higher-order sensorimotor integration

Loss of sensory feedback is a key component of post-stroke sensorimotor loop disruption. It affects touch, position sense, kinesthesia, movement monitoring, error correction, and motor learning. Longitudinal subacute studies show that hand proprioception and motor function do not always recover in parallel, suggesting that sensory and motor recovery may follow partly distinct trajectories (Zbytniewska-Mégret et al., 2023). In late subacute and chronic stroke, fine hand function is influenced by finger proprioception, individuation, force release, and rhythmic control, and technology-based assessments can capture functional variance that conventional clinical scales may overlook (Van Ravestyn et al., 2024). Meta-analytic evidence supports a moderate association between proprioception and motor function, indicating that sensory impairment should be considered in recovery assessment and patient stratification (Usher et al., 2025).

Higher-order sensorimotor integration deficits may further limit the use of sensory feedback for movement correction. Stroke can alter tactile and body-centered spatial representations of the hand, reducing the ability to localize sensory events and use them for motor planning (Dupin et al., 2024). In post-stroke limb apraxia, a prolonged temporal window for sensorimotor integration suggests impaired detection of congruence between action and sensory consequences (Nobusako et al., 2025). Quantitative proprioceptive threshold testing may help identify perceptual deficits that are not captured by coarse bedside examination (Hoh et al., 2025).

### 2.3 Interhemispheric imbalance and compensatory network reorganization

Post-stroke sensorimotor loop disruption involves dynamic bilateral reorganization of motor networks, which may support recovery or lead to inefficient compensation. Neuroimaging evidence indicates that these changes extend beyond perilesional tissue and evolve from early local disconnection to bilateral reorganization and later redistribution of functional connectivity (Yu et al., 2024). M1–M1 interhemispheric connectivity may support basic motor control and recovery, particularly when ipsilesional corticospinal tract integrity is compromised (Paul et al., 2023b). However, the classical interhemispheric competition model does not explain all recovery trajectories. State-dependent interhemispheric inhibition may be more informative than resting-state measures, and longitudinal TMS evidence has not confirmed a stable maladaptive role of contralesional transcallosal inhibition in mild-to-moderate subacute stroke (Mirdamadi et al., 2023; Fokas et al., 2025).

The functional significance of compensatory reorganization depends on whether it supports effective movement rather than merely increasing activation or connectivity. Dynamic connectivity and TMS interference studies indicate that the contralesional M1 and anterior intraparietal regions have time- and region-specific roles during post-stroke movement (Hensel et al., 2025). Recovery also involves interactions between motor and non-motor networks, and early inter-network connectivity may predict later recovery trajectories (Lee and Kim, 2025; Zhang et al., 2025b). Contralesional recruitment may therefore be supportive or inefficient depending on lesion severity and task demands (Tam et al., 2024; Suputtitadda et al., 2025). Brain aging and neurodegenerative changes may further influence upper-limb outcomes by reducing structural reserve and use-dependent plasticity (Takyi et al., 2025).

Subcortical and cerebellar circuits also contribute to sensorimotor relay, movement timing, error correction, and motor learning after stroke (Baker et al., 2023). The dentato-thalamo-cortical pathway links cerebellar output with motor and premotor cortices and may influence cortical excitability during recovery (Li et al., 2024). Dentate nucleus deep brain stimulation combined with rehabilitation has shown feasibility in chronic stroke, while cerebellar non-invasive stimulation may improve selected motor outcomes, although protocols and responder profiles remain heterogeneous (Liu et al., 2024).

## 3 Principal mechanisms through which closed-loop BCI may facilitate post-stroke functional recovery

**Figure 2:**
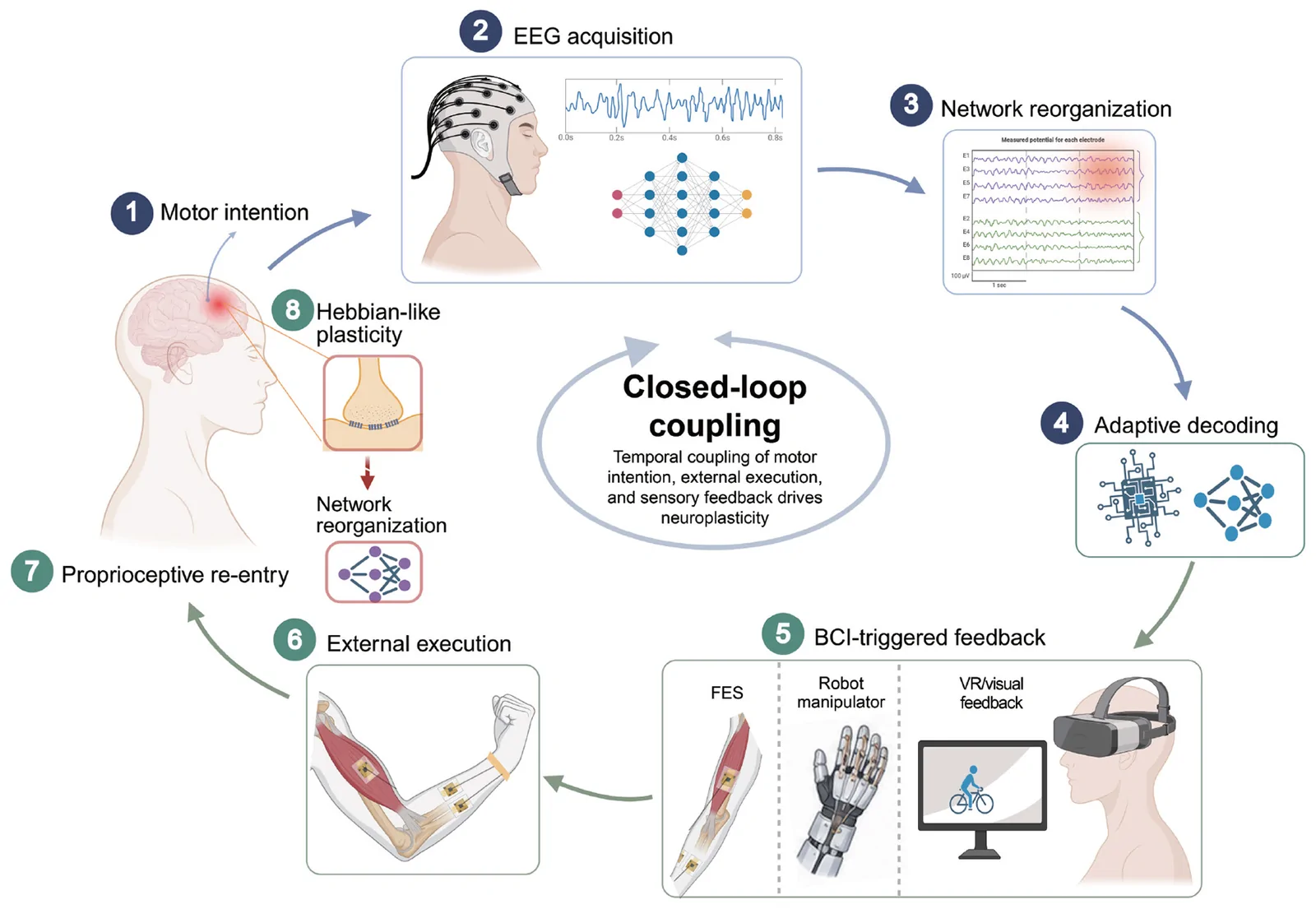
Conceptual schematic of putative mechanisms through which closed-loop BCI may facilitate post-stroke functional recovery. Closed-loop BCI detects residual motor intention, triggers feedback or assistance according to the patient's neural state, and couples intention with peripheral execution and sensory feedback. The figure summarizes motor intention detection, state-dependent triggering, intention-execution-feedback coupling, sensorimotor re-entry, and network remodeling. These pathways summarize the mechanisms currently considered most plausible on the basis of available evidence, although their causal roles have not yet been established experimentally.

The therapeutic logic of closed-loop BCI can be summarized as motor-related brain-state detection, contingent feedback, and potential plasticity induction. As shown in Figure 2, motor intention recognition and state-dependent triggering are the most directly supported components, as human studies have shown that motor imagery, motor attempt, and sensorimotor rhythms can be decoded to drive feedback or assistance. By contrast, intention-execution-feedback coupling, sensory re-entry, and network remodeling remain proposed downstream mechanisms supported mainly by neurophysiological principles, mechanistic correlates, and indirect clinical evidence (Tonin et al., 2025).

### 3.1 Motor intention recognition and state-dependent intervention triggering

The primary role of closed-loop BCI is to detect residual motor intention despite impaired peripheral motor output and use it for real-time therapeutic triggering. Unlike open-loop training delivered according to a fixed schedule, closed-loop BCI identifies task-related brain states during active participation, commonly through EEG sensorimotor rhythms, event-related desynchronization or synchronization, movement-related cortical potentials, and features related to motor imagery or motor attempt (Tonin et al., 2025; Ma et al., 2025; Fu et al., 2022). Feedback or assistance is delivered when the detected state reaches a predefined or adaptive threshold, allowing therapeutic input to be coupled to ongoing neural activity rather than external timing. This state-dependent contingency distinguishes closed-loop BCI from conventional robotic, electrical stimulation, or virtual feedback training.

The performance of state-dependent triggering depends on decoding accuracy, response latency, signal stability, and individualized adaptation. Recent studies show that non-invasive EEG decoding can recognize intended finger movements with increasing precision under experimental conditions, while longitudinal stroke datasets reveal substantial inter-individual variability and training-related changes in motor imagery signals (Ding Y. et al., 2025; Liu Y. et al., 2025). Adaptive BCI systems may help address low signal-to-noise ratio, signal non-stationarity, and limited BCI control by adjusting classifiers, thresholds, and feedback strategies (Zhang et al., 2023). However, decoded brain states may be influenced by attention, visual strategies, compensatory EMG activity, and artifacts, and training-related ERD changes are not uniform across patients (Lu Y. et al., 2025).

### 3.2 Activity-dependent plasticity induced by intention-execution-feedback coupling

The therapeutic effect of closed-loop BCI is primarily attributed to temporal coupling among motor intention, peripheral execution, and sensory feedback rather than assistance alone. Real-time detection of motor attempt or imagery can trigger functional electrical stimulation, mechanical assistance, or embodied feedback, aligning central motor activity with afferent input and potentially supporting Hebbian-like plasticity (Krueger et al., 2024; Biasiucci et al., 2018). Post-stroke recovery also involves changes in corticospinal excitability, sensorimotor integration, interhemispheric balance, attentional networks, and recruitment of spared circuits (Campos et al., 2023). Systematic reviews and meta-analyses suggest that BCI-FES is associated with improved upper-limb function, although effects vary with treatment timing, stimulation parameters, feedback modality, and stroke stage (Liang et al., 2026). Closed-loop stimulation may also provide tactile, proprioceptive, or cortical neuromodulatory feedback through peripheral stimulation, TMS, tDCS, or tACS (Tang et al., 2024; Zhang J. J. Y. et al., 2025).

Clinical evidence supporting this mechanism is promising but incomplete. Randomized studies suggest that motor imagery-based BCI combined with conventional rehabilitation can improve upper-limb function and attention-related performance, with some studies reporting functional gains accompanied by enhanced brain activity (Liu et al., 2023; Liao et al., 2023). Graph-theoretical analysis further suggests that BCI training may modulate visual and dorsal attention networks during upper-limb recovery, indicating involvement of task monitoring, visuospatial attention, and motor planning beyond the primary motor cortex (Ma et al., 2023). A recent randomized trial combining motor imagery and motor attempt, with EEG, EMG, and fNIRS assessments, also supports the potential influence of BCI training on both motor output and cognitive-motor network states (He et al., 2025).

### 3.3 Sensorimotor re-entry-driven local circuit reinforcement and brain network remodeling

A further proposed mechanism is sensorimotor re-entry and task-related network remodeling. When decoded motor intention triggers hand opening, grasping, tactile stimulation, or visual action feedback, proprioceptive, tactile, and visual inputs re-enter sensorimotor networks in a manner aligned with active intention. Clinical and feasibility studies suggest that upper-limb BCI training may improve motor performance and may be accompanied by changes in ipsilesional cortical activity, EEG rhythms, or structural-functional indices (Cantillo-Negrete et al., 2025). In subacute stroke, fNIRS evidence from BCI-controlled soft robotic gloves suggests that intention-driven peripheral assistance may modulate sensorimotor cortical activation (Ji et al., 2025). Multisensory BCI integrating proprioceptive, tactile, and visual feedback may facilitate recovery-related network reorganization by strengthening sensory re-entry and higher-order sensorimotor integration (Lu R. et al., 2025). These neurophysiological changes should be interpreted as mechanistic correlates associated with recovery rather than as direct evidence that network remodeling causes functional improvement.

At the systems level, BCI-related network changes are better understood as changes in task-relevant network efficiency rather than nonspecific activation expansion. BCI combined with functional electrical stimulation has been associated with interhemispheric resting-state connectivity changes that correlate with motor improvement (Sinha et al., 2021). Meta-analytic evidence from randomized trials supports the potential benefit of BCI for post-stroke motor recovery, but effect sizes vary, and patient selection and training protocols remain heterogeneous (Mortezaei et al., 2026). SSVEP-based BCI control of a soft robotic glove may improve engagement and feedback accessibility in patients with poor motor imagery capacity, although its coupling with motor cortical output may be weaker than motor imagery or motor attempt paradigms (Guo et al., 2022).

## 4 Major implementation modes of closed-loop BCI and their synergistic mechanisms

**Figure 3:**
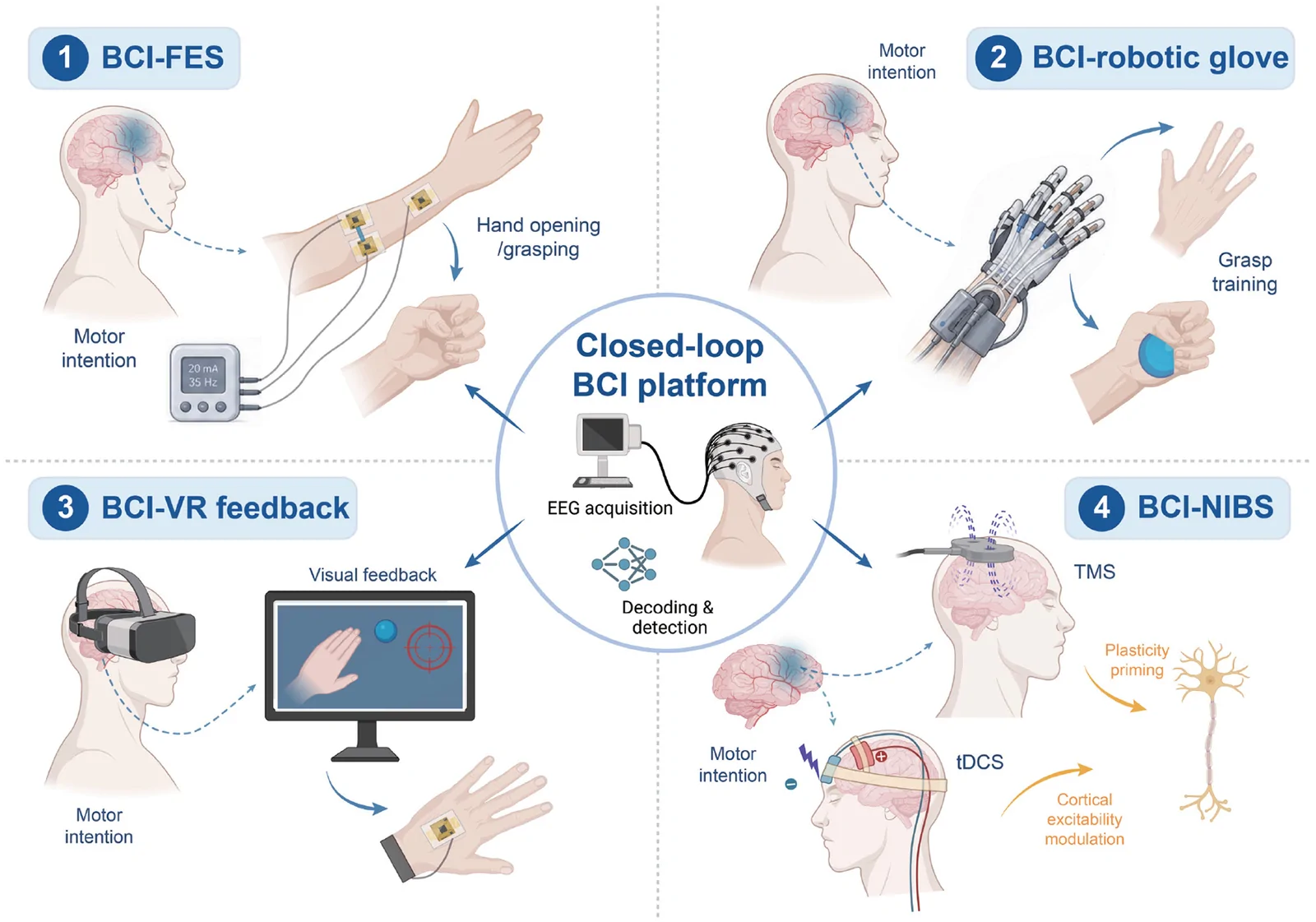
Conceptual schematic of major implementation modes of closed-loop BCI. Closed-loop BCI can be combined with functional electrical stimulation, robotic or soft-actuator assistance, virtual reality and multisensory feedback, or non-invasive brain stimulation. These modes share the principle of intention-contingent feedback but differ in technical configuration, dominant therapeutic rationale, and evidence maturity. The therapeutic rationales shown are conceptual and vary in the strength of supporting clinical and mechanistic evidence.

Closed-loop BCI can be delivered through peripheral, virtual, or neuromodulatory interfaces, but its therapeutic value depends on intention-contingent feedback. Figure 3 summarizes the main implementation modes: BCI-FES supports intention-matched muscle activation, BCI-robot systems provide task-specific assistance, BCI-VR and multisensory feedback enhance embodiment and engagement, and BCI combined with non-invasive brain stimulation aims to modulate cortical excitability during training.

### 4.1 BCI combined with functional electrical stimulation

BCI combined with functional electrical stimulation is the implementation mode that most directly links central motor intention with peripheral movement. In this paradigm, EEG features related to motor imagery or motor attempt are decoded to trigger stimulation of target muscles, producing intention-matched contraction, joint movement, and proprioceptive reafference (Zhang et al., 2025a). Compared with conventional FES, the key feature of BCI-FES is the temporal coupling between the patient's brain state, muscle activation, and sensory feedback. Systematic and network meta-analytic evidence suggests that BCI-FES may improve post-stroke upper-limb Fugl-Meyer scores more than conventional rehabilitation or FES alone, although stimulation parameters, threshold strategy, total dose, and stroke stage may influence outcomes (Zhang L. et al., 2025). Motor imagery-based BCI-controlled electrical stimulation has also been tested for lower-limb recovery in acute hemiplegic stroke, suggesting potential cross-limb applicability, but decoding tasks and outcome domains require separate validation (Luo X. et al., 2024).

### 4.2 BCI combined with robots or soft actuators

BCI combined with robots or soft actuators converts decoded motor intention into controllable and repeatable limb assistance, enabling task-specific practice in patients with severe paresis. Systematic evidence suggests that BCI-robot training may improve post-stroke upper-limb function, but its inconsistent advantage over robot therapy alone leaves the incremental clinical benefit specifically attributable to intention-contingent BCI control uncertain (Qu et al., 2024). A recent network meta-analysis suggested that BCI-based rehabilitation robots may rank highly for upper-limb improvement, although long-term effects are uncertain and device structures, tasks, and doses vary substantially (Zhou L. et al., 2025). Soft robotic gloves provide a lightweight and wearable platform closer to daily grasping tasks, and hybrid BCI-controlled glove studies suggest that multimodal control may improve intention detection and assistance accessibility (Guo et al., 2022; Zhang et al., 2024). However, BCI-controlled exoskeleton and robotic systems still face challenges related to comfort, latency, active engagement detection, and the cost of home deployment (Colucci et al., 2022; Boardsworth et al., 2025).

### 4.3 BCI combined with virtual reality, multisensory feedback, and peripheral sensory stimulation

BCI combined with virtual reality and multisensory feedback can enhance visualization, embodiment, and ecological relevance of motor intention. VR converts motor imagery or attempted movement into immediate visual action feedback, while multisensory feedback adds tactile, proprioceptive, auditory, or action-observation information to approximate real-world sensory input (Oliveira et al., 2025; Mani Bharathi et al., 2024). Compared with unimodal visual feedback, these systems may better link motor intention, attention, and sensory prediction, especially in patients with limited overt movement but preserved cognitive engagement. Recent studies suggest that MI-based BCI combined with VR may improve attention, executive function, lower-limb performance, or dual-task ability after stroke, indicating possible involvement of cognitive-motor networks beyond motor pathways (Wan et al., 2025a,b). BCI-based engagement feedback in VR may also help regulate training involvement and motor cortical activation, although larger stroke-specific trials are still needed (Lim et al., 2025). Recent meta-analytic and randomized evidence suggests that BCI- and VR-based rehabilitation may improve post-stroke motor recovery (Li et al., 2025; Huang et al., 2024). Peripheral sensory stimulation may further extend this approach by providing low-intensity tactile or proprioceptive input triggered by motor intention, task performance, or movement phase (Alashram, 2026).

### 4.4 BCI combined with non-invasive brain stimulation

BCI combined with non-invasive brain stimulation aims to improve responsiveness to closed-loop training by modulating cortical excitability and plasticity. TMS, tDCS, and tACS can be delivered according to decoded brain states, motor intention, or task phase, while BCI provides task-related feedback when intention is detected (Saway et al., 2024; Safdar et al., 2023). A randomized study found that kinesthetic motor imagery BCI combined with tDCS improved upper-limb function in subacute stroke but did not consistently outperform BCI alone when treatment duration was matched (Ming et al., 2025). A network meta-analysis ranked BCI-FES combined with tDCS favorably, although the small number of trials and substantial heterogeneity limit the strength of this finding (Zhang L. et al., 2025). Functional imaging studies further suggest that tDCS may alter training-related brain activity, but the effects vary with stimulation polarity, target, stroke stage, and residual neural pathways (Hu et al., 2021). These findings indicate that stimulation timing, target, and parameters may need to be adapted to the patient's brain state and task performance.

More broadly, the effects of closed-loop BCI may depend less on the specific feedback device than on the temporal coupling of motor intention, assisted movement, and sensory feedback. Among current implementation modes, BCI-FES most directly reflects this principle and has comparatively stronger clinical support. For other configurations, the additional benefit of BCI control over the paired rehabilitation technology remains uncertain. Table 1 summarizes the representative evidence, clinical stage, outcomes, and limitations of the closed-loop BCI implementation modes discussed in this section.

**Table 1:** Representative evidence and translational status of closed-loop BCI implementation modes in post-stroke rehabilitation.

Modality	Evidence and stage	Therapeutic rationale	Clinical outcomes	Translational gaps	References
BCI-FES	Relatively strong evidence; early clinical translation	Intention-contingent muscle activation and proprioceptive re-entry	FMA-UE, motor function, cortical activation, neuroplasticity-related outcomes	Dose-response relationship, stimulation parameters, latency control, long-term durability	Zhang et al., 2025a; Zhang L. et al., 2025; Luo X. et al., 2024
BCI-robot / soft actuator	Emerging clinical evidence; transitional clinical stage	Decoded intention coupled with task-specific kinematic assistance	Arm-hand function, grasp training, movement quality, task practice	Device heterogeneity, uncertain additive benefit, comfort, cost, home deployment	Qu et al., 2024; Zhou L. et al., 2025; Zhang et al., 2024; Boardsworth et al., 2025
BCI-VR / multisensory feedback	Preliminary evidence; experimental stage	Embodied feedback, multisensory integration, and engagement enhancement	Attention, executive function, balance, task-related feedback, motor engagement	Limited large RCTs, cognitive load, cybersickness, feedback authenticity	Oliveira et al., 2025; Mani Bharathi et al., 2024; Wan et al., 2025a,b
BCI-NIBS	Preliminary mechanistic evidence; experimental stage	Brain-state modulation and plasticity priming during closed-loop training	Cortical excitability, upper-limb function, training-related brain-state modulation	Optimal target, polarity, timing, dose, and responder profile remain unclear	Zhang L. et al., 2025; Safdar et al., 2023; Ming et al., 2025

BCI-FES, brain–computer interface-controlled functional electrical stimulation; BCI-VR, brain–computer interface-based virtual reality; BCI-NIBS, brain–computer interface combined with non-invasive brain stimulation; FMA-UE, Fugl–Meyer Assessment of the Upper Extremity; RCT, randomized controlled trial. References were selected from studies cited in the corresponding subsection.

## 5 Biomarkers for predicting BCI efficacy and patient stratification

### 5.1 Electrophysiological biomarkers and brain-state responsiveness

Electrophysiological biomarkers are central to predicting the efficacy of closed-loop BCI because they directly reflect whether a patient retains decodable, modulatable, and trainable motor-related brain states. Sensorimotor rhythm ERD, interregional synchrony, brain symmetry indices, and band-power features have been associated with upper-limb recovery after stroke, but studies vary substantially in acquisition conditions, task paradigms, analytical metrics, and follow-up time points, preventing a single generalizable threshold (Milani et al., 2022; Ding L. et al., 2025). During MI-based BCI training, early ERD characteristics, modulation capacity, and feedback-learning curves may indicate brain-state responsiveness; however, chronic stroke patients often show reduced lateralization, unstable rhythm modulation, and heterogeneous imagery strategies, so decoding performance and clinical benefit do not always coincide (Gangadharan et al., 2024). Studies of chronic BCI therapy such as IpsiHand further suggest that changes in resting-state theta-gamma coupling may correlate with motor gains, indicating that prediction should consider plasticity trajectories rather than single-session accuracy alone (Rustamov et al., 2025). Corticomuscular coupling may also supplement EEG-only measures by indexing synchrony between cortical drive and peripheral output (Feng et al., 2025). Electrophysiological stratification should therefore shift from static screening to dynamic assessment integrating baseline EEG, early learnability, brain-muscle coupling, and feedback responsiveness.

### 5.2 Imaging and hemodynamic biomarkers of network reorganization potential

Imaging and hemodynamic biomarkers help assess whether patients retain sufficient network capacity for closed-loop BCI-related reorganization. TMS-derived MEPs remain important marker of corticospinal pathway function, but recent studies suggest that their prognostic value is stronger in acute or subacute stroke than in chronic moderate-to-severe impairment, where MEP status may not reliably predict intervention response (Shanks et al., 2025; Yen et al., 2025; King et al., 2025). fNIRS and functional imaging can measure task-evoked cortical hemodynamics, lateralization, and functional connectivity, making them useful for evaluating motor-network modifiability during closed-loop training (Sun et al., 2024). In subacute stroke, fNIRS responses during robotic training have been used to predict upper-limb recovery, and machine-learning models may improve individualized outcome estimation (Zhou et al., 2024). fNIRS-based nomograms also suggest that combining clinical scores with hemodynamic features may outperform clinical measures alone (Liu M. et al., 2025). In chronic stroke, structural-functional connectivity, biomechanical behavior, and network efficiency may explain different aspects of residual motor capacity (Cai et al., 2024). In multisensory BCI, randomized controlled evidence further shows that treatment-related functional connectivity changes are associated with clinical outcomes, suggesting that functional connectivity may serve as both a mechanistic correlate and a potential response biomarker (Kim et al., 2025).

### 5.3 Structural reserve and disconnection-defined recovery boundaries

Structural reserve and disconnection patterns may define the biological limits of closed-loop BCI-induced plasticity. Recent imaging studies suggest that corticospinal tract (CST) lesion load, CST microstructural integrity, and lesion-induced network disconnection are associated with post-stroke motor outcomes (Olafson et al., 2024; Wen et al., 2025; Khalilian et al., 2025). However, no validated neuroimaging threshold can currently be used to exclude patients from closed-loop BCI. These markers should therefore be used for patient stratification rather than as rigid cut-offs (Millot et al., 2024).

### 5.4 Integrated clinical phenotypes and responder stratification

Integrating clinical phenotypes with multimodal biomarkers may improve BCI stratification. Structural connectome-based models may help estimate early upper-limb recovery potential, particularly in severely impaired patients whose outcomes are difficult to predict from clinical measures alone (Park and Kim, 2024). PREP2 combines clinical assessment, age, and MEP status, but its predictive performance may be reduced in patients with cognitive impairment, aphasia, neglect, or atypical recovery trajectories (Millot et al., 2024). Technology-based rehabilitation trials often underrepresent patients with severe paresis, which limits the generalizability of current findings (Colamarino et al., 2024). A meta-analysis of chronic stroke BCI therapy suggests an overall favorable effect, although treatment responses vary across patients and settings (Chen and Yun, 2026). Early clinically meaningful improvement may provide an additional indicator of treatment responsiveness (Prasad et al., 2026). Patient selection should therefore consider corticospinal tract integrity, motor-intention decodability, sensory and cognitive status, and early training response rather than relying on a single biomarker. These factors may support the selection of BCI paradigms, feedback modalities, and training intensity, although their predictive value requires prospective validation, as summarized in Table 2.

**Table 2:** Pragmatic stratification framework for closed-loop BCI selection in post-stroke rehabilitation.

Stratification factor	Key assessment	Clinical implication	References
Stroke chronicity and severity	Stroke stage; baseline motor impairment	Informs training intensity, duration, and expected recovery trajectory	Liu J. et al., 2025; Chen and Yun, 2026; Guo et al., 2026
Corticospinal and structural reserve	MEP status; CST integrity; lesion and disconnection patterns	Guides selection of intention-driven BCI, assisted training, or multimodal paradigms	Olafson et al., 2024; Wen et al., 2025; Khalilian et al., 2025; Millot et al., 2024
Motor imagery and signal decodability	ERD modulation; EEG stability; task-related activation	Supports MI-based, motor attempt-based, adaptive, or hybrid BCI paradigms	Milani et al., 2022; Ding L. et al., 2025; Gangadharan et al., 2024
Cognitive and sensory status	Attention; neglect; fatigue; proprioceptive and tactile function	Informs task complexity, supervision level, and feedback modality	Usher et al., 2025; Hoh et al., 2025; Millot et al., 2024
Early training response	Decoding gain; feedback learning; early motor change	Informs continuation, intensification, or protocol adjustment	Prasad et al., 2026; Rampioni et al., 2025

BCI, brain–computer interface; CST, corticospinal tract; EEG, electroencephalography; ERD, event-related desynchronization; MEP, motor evoked potential; MI, motor imagery.

## 6 Key issues and optimization pathways for clinical translation of closed-loop BCI

Clinical translation of closed-loop BCI requires better matching among residual brain activity, feedback modality, patient characteristics, and rehabilitation settings. The following section discusses the remaining barriers, including patient heterogeneity, intervention timing, dose design, mechanism-linked evidence, and long-term deployment.

### 6.1 Patient heterogeneity and trial-design mismatch

A major translational challenge for closed-loop BCI is the mismatch between patient heterogeneity and trial design. Systematic reviews and meta-analyses support the safety and potential efficacy of BCI-assisted stroke rehabilitation, but enrolled patients vary widely in stroke stage, lesion location, residual motor capacity, sensory impairment, cognition, motor imagery ability, and co-interventions, leading to inconsistent effect sizes and limited generalizability (Liu J. et al., 2025; Mortezaei et al., 2026). Evidence from lower-limb and chronic upper-limb BCI studies further suggests that stroke stage, feedback modality, training dose, and outcome domain contribute to treatment heterogeneity (Chen and Yun, 2026; Guo et al., 2026). Future trials should therefore adopt mechanism-informed stratification based on corticospinal reserve, signal decodability, sensory-cognitive status, and target functional domain.

### 6.2 Lack of standardization in treatment windows, dosing, and outcome measures

Unstandardized intervention dose, treatment window, and outcome selection remain major barriers to evidence synthesis and clinical prescription. The therapeutic window for closed-loop BCI should be defined by stroke stage, while optimal timing, intensity, frequency, and schedule remain uncertain (O'Flaherty et al., 2024). Early and subacute intervention may be particularly relevant because residual motor intention can be paired with contingent feedback during a period of greater recovery potential, whereas chronic-stage rehabilitation should be guided by residual pathway integrity, brain-state decodability, sensory-cognitive status, and early response to feedback (Brunner et al., 2024). Dose should include not only total training time and number of sessions, but also successful closed-loop triggers, decoding accuracy, feedback latency, task difficulty, active engagement time, and home-practice adherence (Alt Murphy et al., 2025; Gauthier et al., 2024). Recent successful protocols commonly combine repeated BCI sessions with conventional rehabilitation and contingent feedback. A multicenter RCT showed that 1 month of BCI training added to conventional rehabilitation improved FMA-UE scores in ischemic stroke, while a double-blinded chronic-stroke RCT showed that motor imagery-contingent BCI-FES improved wrist strength, active range of motion, and EEG connectivity compared with non-contingent feedback (Wang et al., 2024; Kim et al., 2025). Outcome selection should be standardized to include FMA-UE, ARAT or Box and Block Test, Motor Activity Log, safety outcomes, decoding accuracy, successful closed-loop triggers, feedback latency, and neurophysiological responsiveness (Teasell et al., 2025; Mehrabi et al., 2025).

### 6.3 Insufficient coupling between mechanistic and efficacy evidence

A further translational limitation is the weak alignment between mechanistic and efficacy evidence. Many studies report improvements in Fugl-Meyer scores, ARAT, or activities of daily living without concurrently reporting decoding quality, effective closed-loop triggering, corticomuscular coupling, cortical activation, or functional connectivity (Jin et al., 2024). Adaptive BCI studies emphasize classifier updating, threshold adjustment, and individualized feedback, but these algorithmic indicators are rarely included as clinical trial endpoints (Tang et al., 2026). Portable adaptive BCI and real-time stimulation systems suggest that brain-state monitoring, neuromodulation, and behavioral training can be integrated into dynamically regulated therapeutic systems, although randomized validation remains limited (Prasad et al., 2026). Real-world BCI data further indicate that some chronic stroke patients may require prolonged use to achieve clinically meaningful improvement, suggesting individualized response trajectories (Rampioni et al., 2025). To improve comparability, future closed-loop BCI trials should report standardized clinical outcomes, neurophysiological biomarkers, BCI performance metrics, dose parameters, and follow-up assessments. A proposed reporting framework is summarized in Table 3.

**Table 3:** Minimum reporting items for future closed-loop BCI trials in post-stroke rehabilitation.

Reporting domain	Minimum reporting items
Clinical outcomes	FMA-UE, ARAT, or other motor scales; activity-level outcomes; adverse events; responder definition; follow-up duration
Neurophysiological markers	EEG/ERD features; fNIRS activation; TMS/MEP status; EMG or corticomuscular coupling; functional connectivity when available
BCI performance	Decoding accuracy; trigger success rate; false activation rate; feedback latency; calibration time; signal stability
Treatment dose	Total training time; number of sessions; task repetitions; successful closed-loop activations; stimulation parameters; feedback modality
Follow-up and implementation	Short- and long-term follow-up; adherence; usability; withdrawal reasons; home training or remote monitoring data when applicable

ARAT, Action Research Arm Test; BCI, brain-computer interface; EEG, electroencephalography; EMG, electromyography; ERD, event-related desynchronization; FMA-UE, Fugl-Meyer Assessment of the Upper Extremity; fNIRS, functional near-infrared spectroscopy; MEP, motor evoked potential; TMS, transcranial magnetic stimulation.

### 6.4 Home deployment, adaptive closed loops, and long-term follow-up

Home deployment is essential for improving access to closed-loop BCI rehabilitation, but it requires systems that are safe, usable, minimally burdensome, and remotely monitorable. Reviews of digital upper-limb rehabilitation show that telerehabilitation, gamification, virtual reality, sensors, mobile technologies, and robotics are increasingly used at home; however, current evidence remains focused mainly on feasibility, acceptability, and short-term outcomes, with limited data on long-term effectiveness and real-world adherence (Gebreheat et al., 2024; Kheirollahzadeh et al., 2025). For home-based BCI, technical barriers include EEG setup stability, artifact control, automated calibration, feedback latency, device reliability, and remote troubleshooting (Oliveira et al., 2025). Clinical implementation also depends on patient adherence, caregiver involvement, therapist-guided task selection, usability, and sustained training support, which have been emphasized in participatory design and digital rehabilitation implementation studies (Jarvis et al., 2024; Schopp et al., 2026). Regulatory and data-related issues, including privacy protection, secure data transmission, adverse-event reporting, device maintenance, and responsibility for remote monitoring, should also be addressed before large-scale deployment. A staged implementation pathway could begin with supervised clinic-based calibration, followed by caregiver and therapist training, hybrid clinic-home practice, remote monitoring with technical support, and long-term evaluation in multicenter studies.

## 7 Conclusions and perspectives

A major challenge in post-stroke sensorimotor recovery is not only insufficient motor output, but also disruption of the closed loop linking motor intention, corticospinal transmission, sensory feedback, higher-order sensorimotor integration, and bilateral network regulation. This review summarized the neurobiological basis of this disruption, including impaired execution of motor intention after corticospinal tract injury, limited error correction due to sensory feedback loss, and the dual role of interhemispheric imbalance and compensatory network reorganization in shaping recovery trajectories. On this basis, closed-loop BCI can detect residual motor intention in real time and couple it with functional electrical stimulation, robotic assistance, virtual reality, multisensory feedback, or non-invasive brain stimulation. By temporally aligning central motor-related activity, peripheral execution, and sensory re-entry, these systems may contribute to activity-dependent plasticity and network remodeling, although the underlying causal mechanisms remain incompletely established.

Closed-loop BCI is an emerging approach to post-stroke sensorimotor rehabilitation, but its clinical maturity varies across implementation modes. The strongest clinical support is currently available for BCI-FES, whereas BCI-robot, BCI-VR, BCI-NIBS, sensory-feedback stimulation, adaptive BCI, and home-based systems remain at more preliminary or transitional stages of evidence. Future research should first confirm the durability and clinical relevance of treatment effects through standardized multicenter trials, and then validate practical criteria for matching patients to appropriate BCI paradigms.
